# Knowledge, Attitude and Practice of Hepatitis B Virus Infection among Dental Students and Interns in Saudi Arabia

**DOI:** 10.4317/jced.54418

**Published:** 2018-01-01

**Authors:** Hashem-Motahir Al-Shamiri, Fadyah-Eid AlShalawi, Tahani-Mohammed AlJumah, Maha-Mohammad AlHarthi, Esraa-Mohammed AlAli, Hanan-Mohammed AlHarthi

**Affiliations:** 1 Department of Oral and Maxillofacial Surgery, Alfarabi colleges, Riyadh, Saudi Arabia; 2 Department of Internship, Alfarabi Colleges, Riyadh, Saudi Arabia

## Abstract

**Background:**

Hepatitis B virus (HBV) is a worldwide healthcare problem. Dental health care professionals are at a high risk of infection by HBV. The present study investigated the knowledge, attitude and practice of HBV infection among Saudi dental students and Interns in Saudi Arabia.

**Material and Methods:**

This was a questionnaire-based cross-sectional study. A self-administered questionnaire consisting of questions on students’ knowledge, attitudes, and practices regarding HBV was used. Data of 420 participants were analyzed using SPSS (Statistical Package for Social Studies) version 22.0.

**Results:**

The response rate was 84%. Overall, the participants showed fair level of knowledge about HBV, with significant differences between final year students and the interns. Also, the subjects showed negative attitude toward HBV patients. The vast majority reported always wearing gloves (97.9%), gowns (92.1%), face masks (89.2%), disposable caps (87.1%) and protective eye wear (80.9%). The majority of participants (91.4%) had been vaccinated against HBV. However, only 41% completed the recommended 3 doses of the vaccine.

**Conclusions:**

These unsatisfactory findings emphasize the necessity of continued education about HBV in order to improve knowledge, attitudes, and practices of dental students and Interns regarding HBV.

** Key words:**HBV, Knowledge, Practice, Dental students, Interns.

## Introduction

HBV infection is a worldwide health care problem, especially in developing countries. It is one of the most common chronic viral infections that may infect the population. About 2 billion people are estimated to be infected and more than 350 million are chronic carriers of the virus (1). A wide spectrum of liver diseases can be caused by HBV infection, these diseases ranging from acute hepatitis to chronic hepatitis, liver cirrhosis, and hepatocellular carcinoma (2). HBV has several modes of transmission like through contact with infected blood or semen, from infected mothers to their neonates. In addition, it can be transmitted through the use of unsafe injections, blood transfusion, or dialysis (1). Even though it is unethical and illegal to refuse treatment of HBV patients, some dentists may deter from treating HBV-positive patients (3).

Dental health care professionals including dental practitioners are at a high risk of infections by various microorganisms like HBV and HCV, herpes simplex virus, HIV, mumps, influenza, and rubella (4). This risk may be accentuated by accidental injuries during patient treatment (5), thus a culture of safety precaution and the infection control practice should be implemented among those students. This culture is the responsibility of dental schools in providing the adequate infection control measures, and training of dental students how protecting themselves and their patients, as well foundation of safe working conditions (6).

A number of studies (3,5-8) worldwide have evaluated the level of dentists’ and dental students’ knowledge, attitudes and behaviors regarding infection control guidelines and precautions and found unsatisfactory response and emphasized the need for further improvement of this kind of knowledge and practice. In Saudi Arabia data on HBV knowledge and attitudes of dental students are lacking. Therefore, this study was conducted to investigate the knowledge, attitudes and practices regarding HBV infection among dental students and Interns, in Riyadh, Saudi Arabia.

## Material and Methods

This study, conducted in March 2017, consisted of a cross-sectional survey of final dental students and Interns. This survey was applied at four dental schools that were: Princess Nora university, Private Al-Qassim university, Riyadh dental college and Alfarabi college of dentistry. The study was approved by Alfarabi college Institutional Ethical Review Board.

The questionnaire was adapted from pretested questionnaires that have been used in some similar studies (7,8). Before distributing the questionnaire, a random sample of students (n=40) to ensure understandability and clarity of the questions.

This self-administered questionnaire consisted of 22-closed-ended questions divided into four parts. The first part screened the demographic profile of students including, age, gender, and academic level. The second part assessed the knowledge of those students regarding HBV infection and routes of transmission. The third part investigated the behavior and attitude towards HBV infection and infected patients. The last part examined the practices of students regarding protection measures against HBV as well as their HBV vaccination status.

Students were asked to fill out the anonymous self-administered questionnaire at the end of the lectures, or during the clinical sessions. Students who agreed to participate in the study, signed a consent form prior to answer the questionnaire.

SPSS (Statistical Package for Social Studies) version 22.0 (IBM Corporation, Chicago, IL, USA) was used for data entry and analysis. Descriptive statistics including frequencies and proportions were performed. Chi-squared test was used to assess statistical significance, with a *p*-value < 0.05 was considered statistically significant.

## Results

The response rate was 84% (420 out of 450). Female students represented about 66.2% of the sample. The sample comprised an almost equal distribution of interns (53.5%) and final year dental students (46.7%). Almost two thirds of the sample were from private dental schools ( Table 1).

**Table 1:**
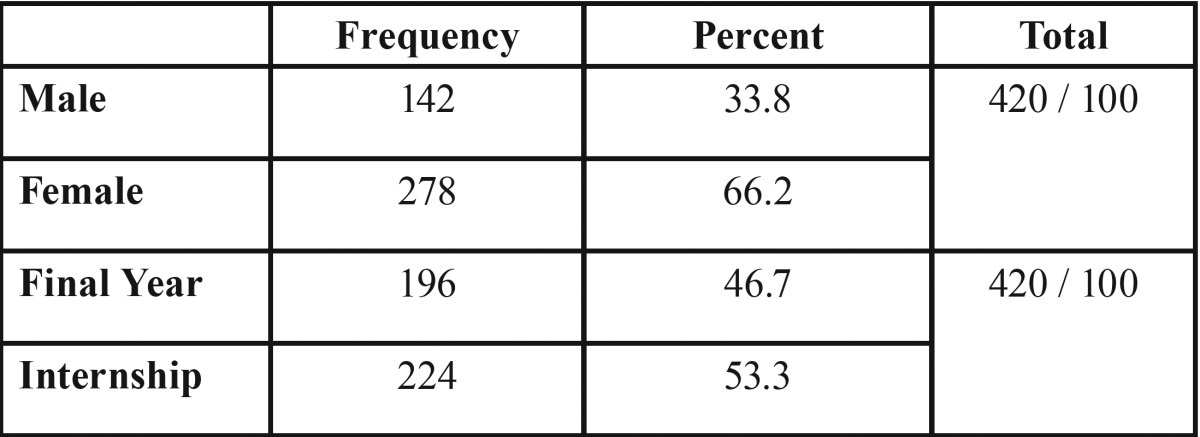
Demographic Distribution (Gender & Academic level ).

 Table 2 shows proportions of “correct” responses to the knowledge items. Overall, correct responses varied greatly from 21% - 92.1%, with significant differences between interns and final year students. The vast majority knew that HBV can be transmitted from patient to patient (89.5%), that HBV can be transmitted through dental treatment (90.7%) and that the HBV vaccine is safe (92.1%). On the other hand, only less than quarter of the subjects knew about HBV sensitivity to low temperature, dryness and ultraviolet rays (21%), and that HBV is not less transmissible than Human Immunodeficiency Virus (HIV) (26%). Overall, final year students were significantly more knowledgeable than intern (*p*<0.05). Regarding gender, there were significant differences between males and females in most of the answered knowledge items. For example, significantly more females answered correctly some HBV knowledge items than their male counterparts: transmission of HBV through saliva (50.9% vs. 26.1%), transmission from dentists to patients (71.6% vs. 59.9%), transmission from patient to patient (91.4% vs. 85.9%) and transmissibility of HBV in comparison to HIV (29.9% vs. 18.3%). On the other hand, significantly more males were more knowledgeable than females regarding infection capacity of HBV outside the body (33.1% vs. 19.8%) and the ability of HBV to survive on unsterilized surfaces (85.9% vs. 76.3%).

**Table 2:**
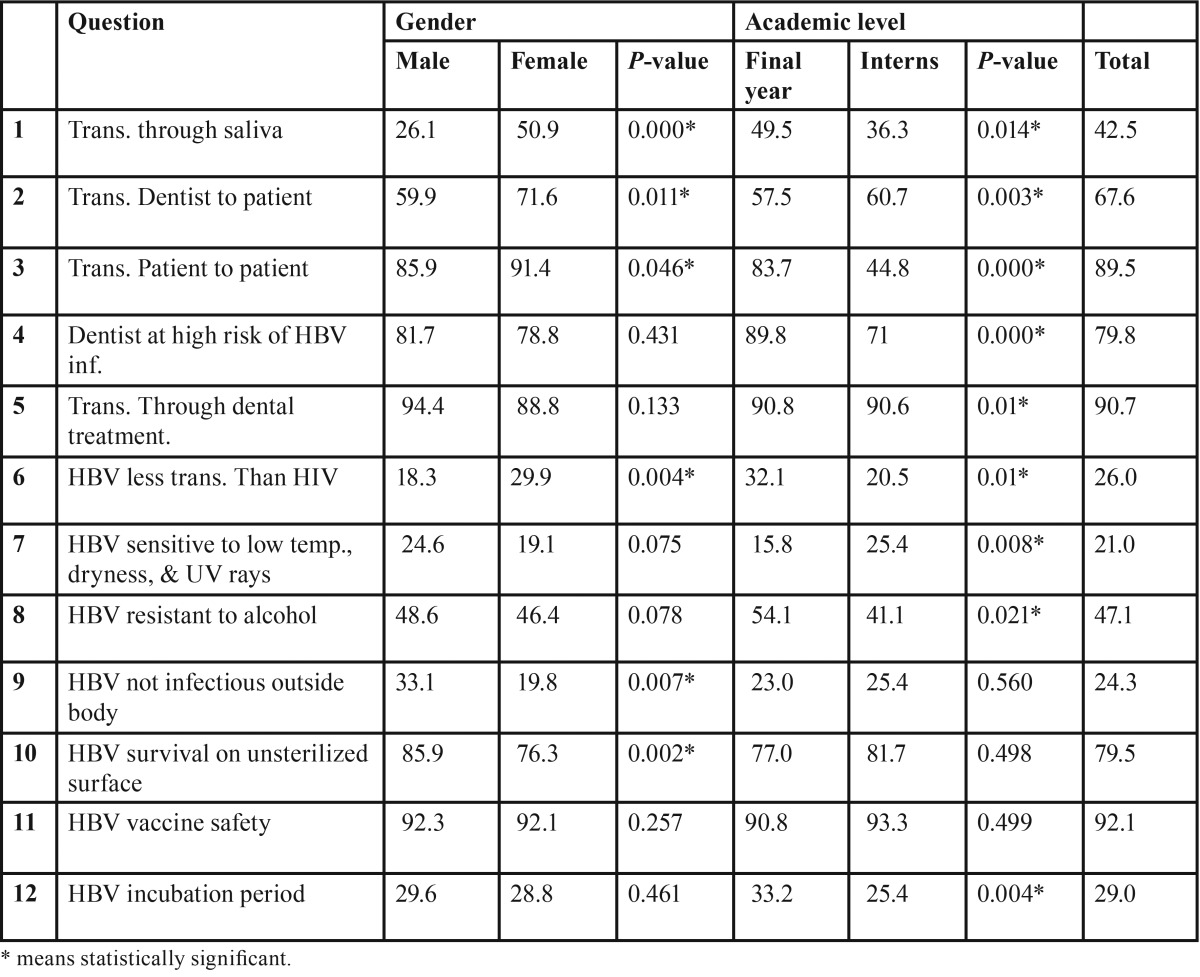
Knowledge regarding Hepatitis B Virus Infection - %.

Regarding the possible modes for HBV transmission ( Table 3), the correct answers ranged between 13.3% and 98.1% with no significant differences between final year students and the interns except in one item related to the possibility of HBV transmission through saliva, in which the final year students answered better than interns (17.3% vs. 15.6%). With reference to gender, there was also no significant differences between males and females except in two items; the first one is related to the possibility of HBV transmission through sexual route, favoring males over females (76.3% vs. 69.7%). The other one is the transmissibility of HBV through saliva, favoring females over males (18.3% vs. 15.5%).

**Table 3:**
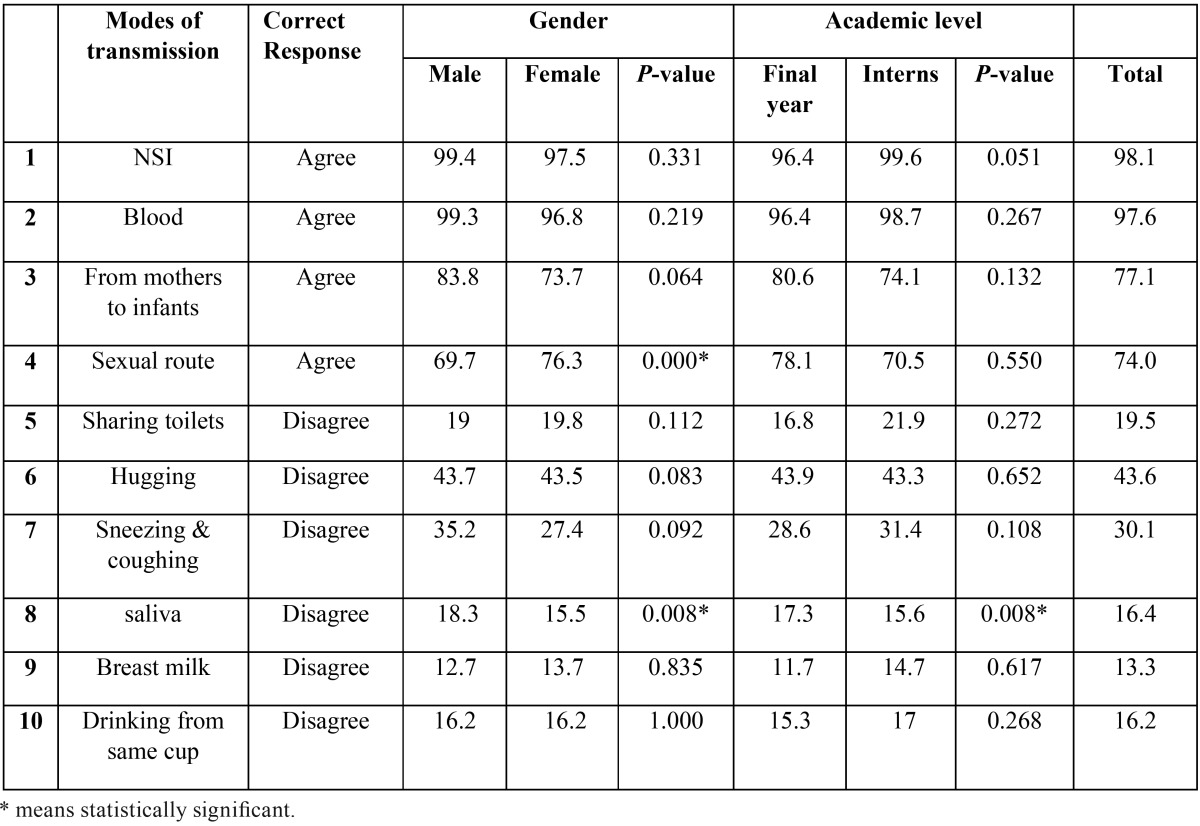
Knowledge regarding Hepatitis B Virus Modes of Transmission – %.

 Table 4 describe the attitude of the students towards patients with HBV. Overall the attitude of the participants was unsatisfactory and showed a negative attitude toward HBV infected patients. Females, significantly showed better attitude towards HBV patients than males. Also, final year students showed better attitude than their counterpart interns.

**Table 4:**
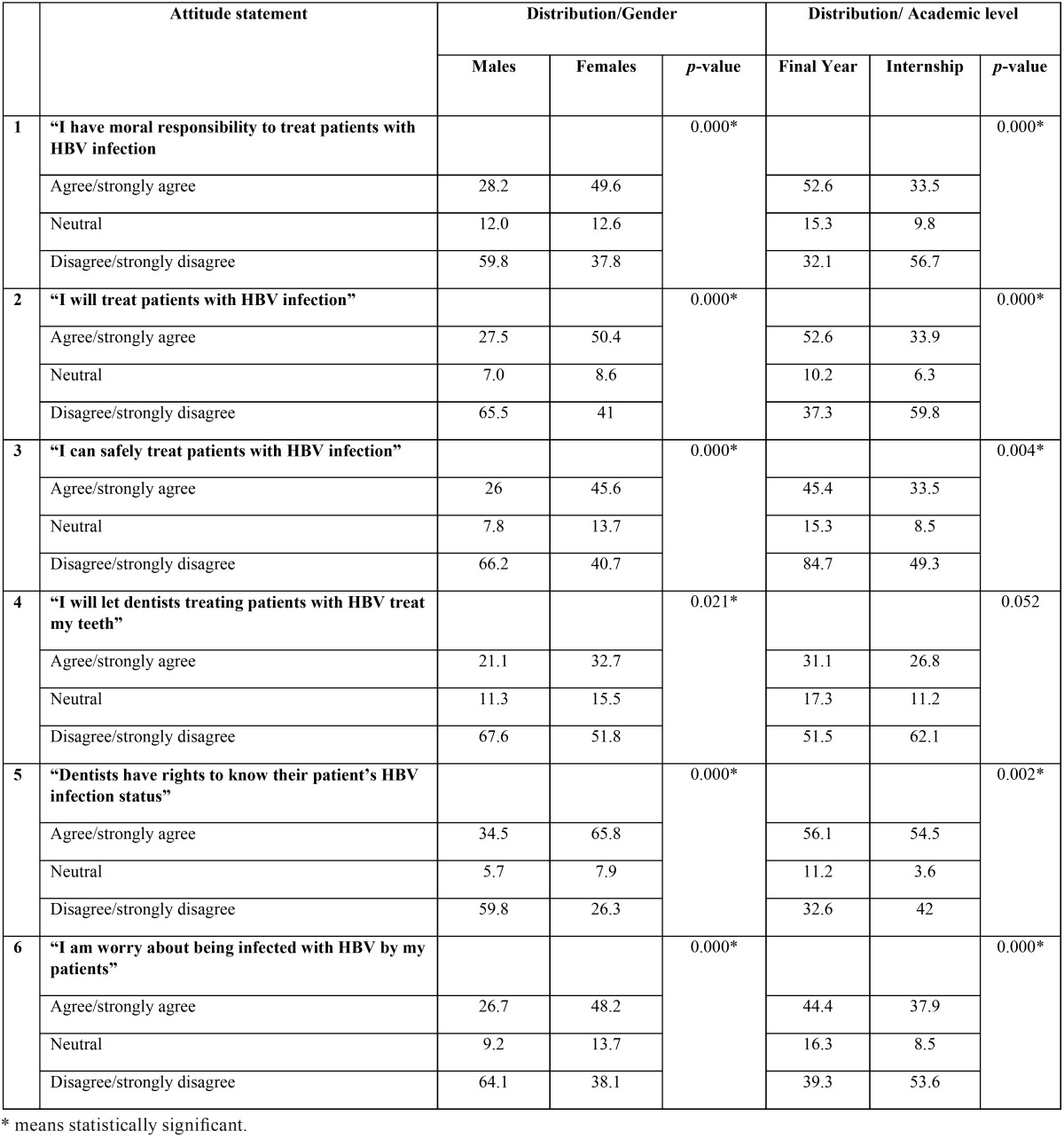
Attitude toward Hepatitis B Virus Infection - %.

 Table 5 shows students’ use of personal protective equipment. The vast majority reported always wearing gloves (97.9%), gowns (92.1%), face masks (89.2%), disposable caps (87.1%) and protective eye wear (80.9), with no significant differences according to gender or the academic year. Significantly more females reported always wearing disposable caps than male students (93.7% vs. 83.8%).

**Table 5:**
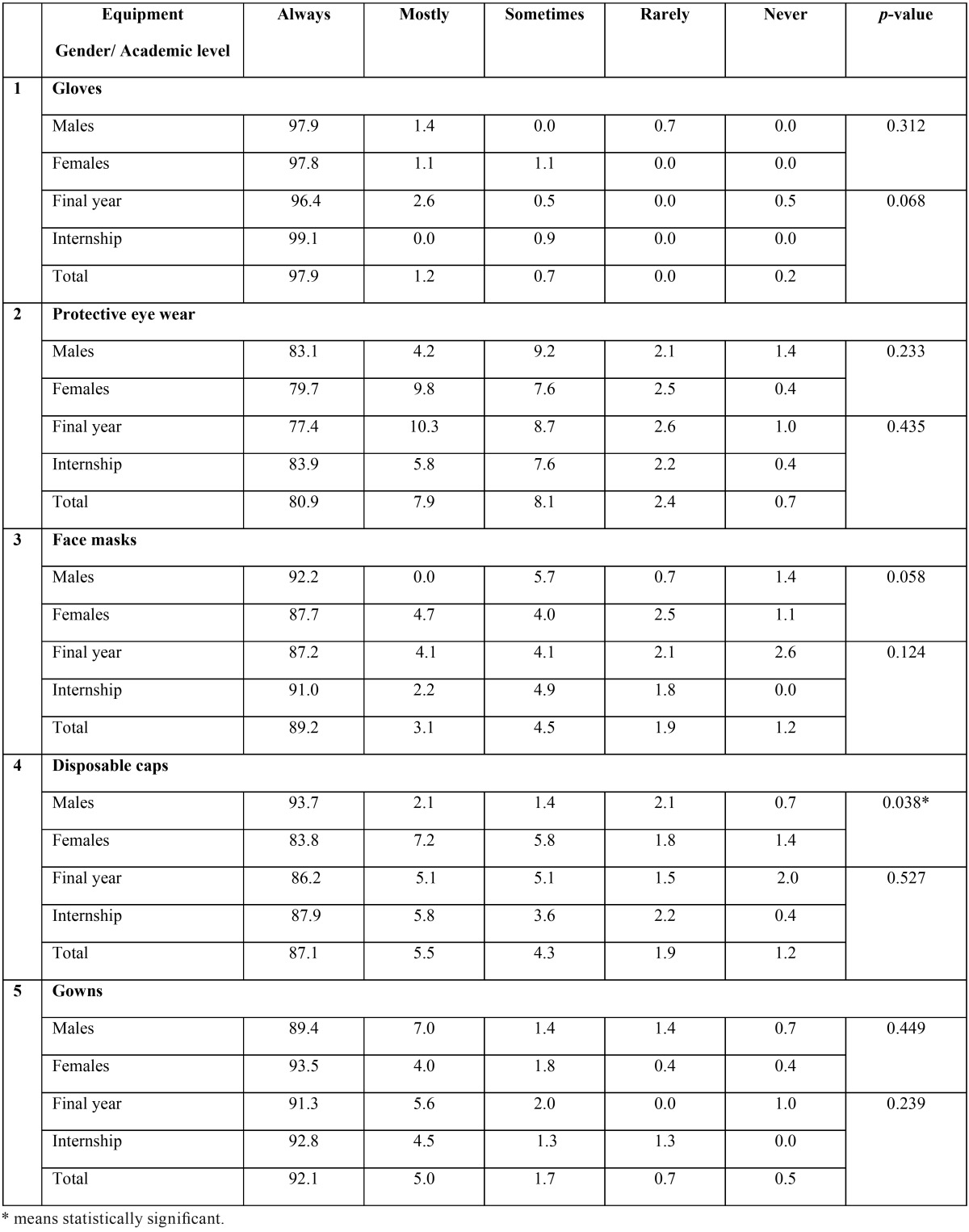
Student’s use of Personal Protective Equipment - %.

 Table 6 shows the vaccination status of the participants. Vaccination was reported by almost 91.4% of the participants, with a significant difference between males and females (88.6% vs. 93.9%). Out of the vaccinated participants, only 41% completed the recommended 3 doses of vaccination with significant difference according to the academic level (*p* <0.05). No correlation was found between number of doses and gender.

**Table 6:**
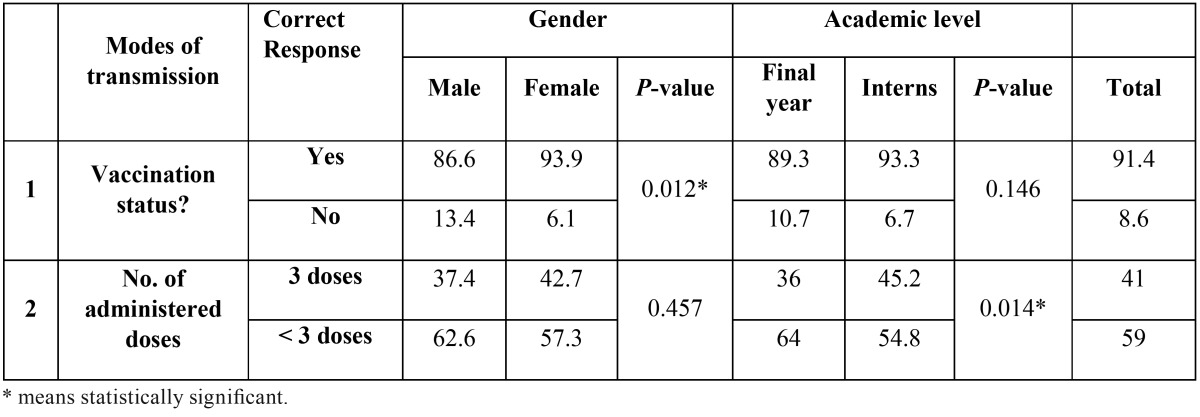
HBV Vaccination – %.

## Discussion

HBV infection is one of the most significant vocational infection that may face dental professionals, as they are frequently exposed to blood, saliva, and even suffer needle stick injuries (9,10). Accordingly, in order to reduce or prevent the transmission of such microorganisms to dental health workers, a strict adherence to infection control guidelines should be applied. This study was conducted to assess the level of knowledge, attitudes, and practice among dental students and interns regarding HBV infection at 4 dental schools in Saudi Arabia.

Overall, dental students in the present study showed poor attitude and fair level of knowledge regarding HBV infected persons. These results were also reported in other studies (7).

Based on the results of this study, we can deduce that Saudi dental students have a fairly unsatisfied level of knowledge regarding HBV infection (only 6 out of 12 items with correct response rave above 50%). This is in an agreement with some other studies that showed limitation in knowledge among health care workers regarding HBV infection and its occupational risk (8,11-14).

In a study conducted by Alavian *et al.* (15), about 81.7% and 98.6% of the participants knew about the possibility of HBV transmission through saliva and from dentist to patient respectively, while in our study the figures were much lower (42.5% and 67.6% respectively. Interestingly, the vast majority of our participants believed that HBV vaccine is safe and effective for all ages (92.1%). This finding is very close to the finding (95.1%) (8) among Saudi dentists in Al Jouf province in. This may reflect the significance of continuous medical education programs in improving the health behaviors among dental health care workers.

In reference to the knowledge about modes of HBV transmission, only 4 items out of 10 got a response above 50%, demonstrating an inadequate level of knowledge, especially among controversial items such as transmission of HBV through saliva, breast milk, sneezing or coughing of an infected person. This could be attributed to the controversy in these points and the absence of concrete evidence (8,16,17). Of the participants, only 19.5% knew that sharing toilets with an infected person could not transmit HBV. This finding is contradicted with results of Al-Hazmi study (61%) (8).

Regarding the statements on the attitude assessment, unfortunately only a small proportion of the participants were inclined to choose “agree” or “strongly agree” which actually reflect unsatisfactory or negative attitude toward HBV infected people. Interestingly, the obtained data showed better significant attitudes of females compared to males, which was in agreement with some other studies in this subject (6,15). This may be accounted to the fact that females are more concerned with infection control guidelines comparing to males (18). On the other hand, the results were contrary to the expectation where final year students showed significant better attitudes than interns in all statements of attitudes. This finding is in agreement with study conducted by Li *et al.* (7) among Chinese dental interns, and contrary to some other studies (6,15). This may be ascribed to that dental interns are more likely exposed to high risk factors of HBV infection like blood and saliva, making them less willing to deal with HBV patients.

In respect to the use of personal protective equipment, the response was adequate and in agreement with some previous studies in this subject (6,7,18,19). In other hand the reported use of protective barriers in this study was higher than in a previous study in Yemen (5) (face masks 89.8% vs 53.8%, and eyewear 80.9% vs 14%). This adequate utilization of protective barriers among the participants in this study may reveal the good practice and habits cultivated once admitted to the dental school.

The finding of the present study indicated a high rate of HBV vaccination. About 91.4% of the participants were vaccinated against HBV, which could be ascribed to the fact that HBV vaccination is obligatory requirement by the dental and medical schools in Saudi Arabia. This rate is comparable to that reported by other studies in UAE (98.8%), Brazil (90.8%) and Canada (100%) and Saudi Arabia (90%) (6,18-20). However, this rate is higher than that reported in other studies reported in Yemen (70.7%) and India (38%) (5,21). This study observed that females administered the vaccine were more than males with significance. This finding is in agreement with some studies conducted in Brazil and Yemen (5,18), and could be attributed to the recognized concern of females toward the preventive measures in general (18).

Unfortunately, out of 91.4% of vaccinated participants, only 41% had received the recommended 3 doses of vaccination. This result is much lower than that reported in some other studies by Alavian *et al.* (15), Kramer *et al.* (22) and De Souza *et al.* (18) in which this rate was more than 80%.

## Conclusions

The unsatisfactory findings from this study emphasize the necessity of continued education about HBV in order to improve knowledge, attitudes, and practices of dental students and Interns regarding HBV.
